# 4K ultra HD technology reduces operative time and intraoperative blood loss in colorectal laparoscopic surgery

**DOI:** 10.12688/f1000research.21297.1

**Published:** 2020-02-11

**Authors:** Giulio M. Mari, Jacopo Crippa, Pietro Achilli, Angelo Miranda, Letizia Santurro, Valentina Riggio, Martino Gerosa, Pietro Ascheri, Giuseppe Cordaro, Andrea T.M. Costanzi, Dario Maggioni

**Affiliations:** 1 General Surgery Department, Desio Hospital, ASST Monza, Desio, MB, Italy; 2 General Surgery Residency Program, University of Milan, Milan, Italy

**Keywords:** Colorectal surgery, laparoscopy, 4K full HD

## Abstract

**Background**: HD systems are routinely used in laparoscopic surgery, 4K ultra HD monitors are mainly available within specialized, high-volume laparoscopic centers. The higher resolution of 4K ultra HD video could upgrade the surgical performance improving intraoperative and post-operative outcomes.

**Methods**: We performed a retrospective comparative analysis of intraoperative parameters and post-operative outcomes in a cohort of patients operated on for elective laparoscopic procedures for colo-rectal cancer during two different time frames: 2017 procedures performed using the Visera Elite full HD technology (® Olympus America, Medical) and the 2018 procedures performed the Visera 4K Ultra HD System (® Olympus America, Medical).

**Results**: There was a statistically significant reduction in operative time in patients operated on with the 4K ultra HD technology compared to HD technology (p < 0.05). Intraoperative blood loss was significantly reduced in patients operated in 2018 (p < 0.05). There were no statistically significant differences in complication rate and postoperative outcomes between the two groups.

## Introduction

Laparoscopic surgery provides several intra- and post-operative advantages such as improved cosmesis, less post-operative pain and faster recovery compared to open surgery ^1^. The development of new technologies has allowed laparoscopic surgery to become faster and safer. In particular, the quality of digital imaging has played a key role in the development of such procedures ^2^. The association between quality of vision and surgical outcome is very tight. Improving the image quality allows the sharpness of laparoscopic surgery to be fully expressed. The clear view of the anatomical landmarks can improve the accuracy of dissection, permitting dissection of the lymphatic and nervous structures, which represent a crucial aspect of colorectal surgery ^3^. Furthermore, the improved visualization of vascular structures and the better definition of adipose tissues may avoid intraoperative bleeding and reduce operative time, resulting in reduced surgical stress for the patient and potentially decreased complication rate ^4^.

Recently, several new imaging technologies, such as three-dimensional (3D)/high-definition (HD) stereovision and high-resolution two-dimensional (2D)/4K monitors have been made available to laparoscopic surgeons ^5^. However, it is still unclear to what extent these technologies can actually improve surgical performance ^6^.

Nowadays, HD systems are routinely used in laparoscopic surgery, while 4K ultra HD monitors are mainly available within specialized, high-volume laparoscopic centers ^7, 8^. The higher resolution of 4K ultra HD video could upgrade the surgical performance by improving depth perception, dissection precision, and bleeding control, but no study has yet provided a good evidence to sustain this finding. For this reason, we designed the present study to compare surgical intraoperative and post-operative parameters in patients operated on for colorectal cancer either with 4K ultra HD technology or a standard HD vision.

## Methods

### Ethical statement

Ethical approval was sought for the present study and the need for approval was waived by the ASST-MONZA ethics committee. According to the ASST-MONZA hospital regulation and to the local ethics committee, retrospective studies which do not involve a clinical intervention do not need to undergo the institutional review board ethical approval process. Written informed consent for reanalysis of the data and revision of clinical images and videos was taken from all the patients at the day of the inpatient visit one week before the surgical intervention date, as per routine practice in our institution ^9^.

### Patients

We performed a retrospective analysis of consecutive adult patients operated on for elective laparoscopic procedures for colorectal cancer during two different time frames: 2017 procedures performed using the Visera Elite full HD technology (® Olympus America, Medical) and the 2018 procedures performed the Visera 4K Ultra HD System (® Olympus America, Medical). Patients were divided into groups according to optic technology used. All the operations were performed in one hospital with the same operating room settings during the study period, except for the optic technology used. Patients were excluded from the analysis if they were submitted to surgery for benign disease, for palliative resections or in an emergency setting.

### Surgical procedures

All procedures in 2017 and 2018 were performed with the same hybrid energy device. Surgical procedures were: laparoscopic right hemicolectomy (LRH) with intra-corporeal anastomosis; laparoscopic left hemicolectomy (LLH) with the anastomosis performed according to the Knight-Griffen technique; laparoscopic rectal anterior resection (LRAR) with the anastomosis performed according to the Knight-Griffen technique and loop ileostomy ^10^. All procedures were performed by four colorectal laparoscopic surgeons, each of them with more than 100 totally laparoscopic procedures involving right colectomies, left colectomies and rectal anterior resections performed before 2017. Operating surgeons were asked to provide feedback on the ability of 4K ultra HD technology to visualize anatomical structures with respect to the HD technology, and regarding the eye fatigue perceived at the end of a procedure with 4K ultra HD technology over the HD technology. Feedback was collected through a written survey that was filled out at the end of each surgical procedure. This survey was routinely completed by the staff surgeons at the end of every minimally invasive procedure as part of the surgical intervention notetaking. The surveys were then retrieved form the electronic medical records.

### Data collection

Data were derived from the prospectively maintained electronic database of medical records of our institution. This database contained demographic information, admission dates and discharge diagnoses of each patient undergoing surgery for colorectal cancer. Electronic medical records contained details of every inpatient hospitalization at our institution, every outpatient visit to the clinic or emergency department, as well as every laboratory result and all data concerning the postoperative course after surgery. Specific items in the database are described in Table 2. Time of surgery, blood loss, conversion rate, as well as complication rate according to Clavien-Dindo (CD) classification, were analyzed ^11^. We also focused on CD complication rate for major complications (CD major or equal to 3).

### Statistical analysis

Descriptive statistics for categorical variables were reported as frequencies (%), continuous variables as mean (standard deviation) or median (interquartile range (IQR)) according to distribution. The Chi-square ^2^ test was used to compare categorical variables. All statistical tests were two-sided and a level of less than or equal to 0.05 was used to indicate statistical significance. Data analysis was performed with Statistical Software for the Social Sciences (SPSS) Advanced Statistics 22 (IBM Software Group, 200 W. Madison St., Chicago, IL; 60,606 USA).

## Results

There were no statistically significant differences in terms of baseline characteristics between groups ^9^. Table 1 describes patient characteristics.

**Table 1.** Patient characteristics.

	Total	2017	2018	p
N	191	94	97	
Male	114	52	62	ns
Female	76	42	34	ns
Median age (range)	68 (31–97)	68 (38–97)	70 (31–94)	ns
Mean BMI ± SD	25.0 ± 3.5	26.0 ± 3.8	25.5 ± 2.9	ns
ASA I	62	32	30	ns
ASA II	85	41	44	ns
ASA III	35	17	18	ns
ASA IV	9	4	5	ns
LRH	58	28	30	ns
LLH	82	38	44	ns
LRAR	51	28	23	ns
Stage I	55	28	27	ns
Stage II	66	31	35	ns
Stage III	61	31	30	ns
Stage IV	9	4	5	ns

BMI, body mass index; SD, standard deviation; ASA, American Society of Anesthesiologists’ physical status classification; LLH, laparoscopic left hemicolectomy; LRH, laparoscopic right hemicolectomy; LRAR, laparoscopic rectal anterior resection.

In 2017, a total of 28 LHRs, 38 LLHs and 28 LRARs were performed, while there were 30 LRHs, 44 LLHs and 23 LRARs in 2018. The overall complication rate according to CD classification was 21.2% (20/94) in 2017 and 17.5% (17/97) in 2018 (p>0.05). The rate of major complications (CD ≥ 3) was 6.3% (6/94) in 2017 vs 4.1% (4/97) in 2018 (p>0.05). In particular, there were no statistically significant differences in anastomotic leak rate, post-operative bleeding rate, mortality rate and readmission rate between groups. Mean surgical time was statistically significantly shorter with 4K ultra HD technology than HD technology (p < 0.05). Intraoperative blood loss was statistically significantly reduced in patients operated on in 2018 (p < 0.05) ( Table 2).

**Table 2.** Intraoperative parameters and short-term outcomes.

	Total	2017	2018	p
N	191	94	97	
Complication rate	37/191 (19.3%)	20/94 (21.2%)	17/97 (17.5%)	ns
CD ≥ 3	10/191 (5.2%)	6/94 (6.3%)	4/97 (4.1%)	ns
Leak rate	7/191 (3.7%)	4/94 (4.2%)	3/97 (3.0%)	ns
P.O. bleeding	3/191 (1.5%)	2/94 (2.1%)	1/97 (1.0%)	ns
Readmission rate	21/191 (11.0%)	10/94 (10.6%)	11/97 (11.3)	ns
Mortality rate	1/191 (0.5%)	1/94 (1.0%)	0/97 (0.0%)	ns
Mean operative time (min) ± SD	199 ± 49.7	209 ± 51.4	192.7 ± 48.3	< 0.05
Mean blood loss (ml) ± SD	43 ± 12	48 ± 15	40 ± 18	< 0.05

CD, Clavien-Dindo; P.O., post-operative; SD, standard deviation.

**Figure 1.**
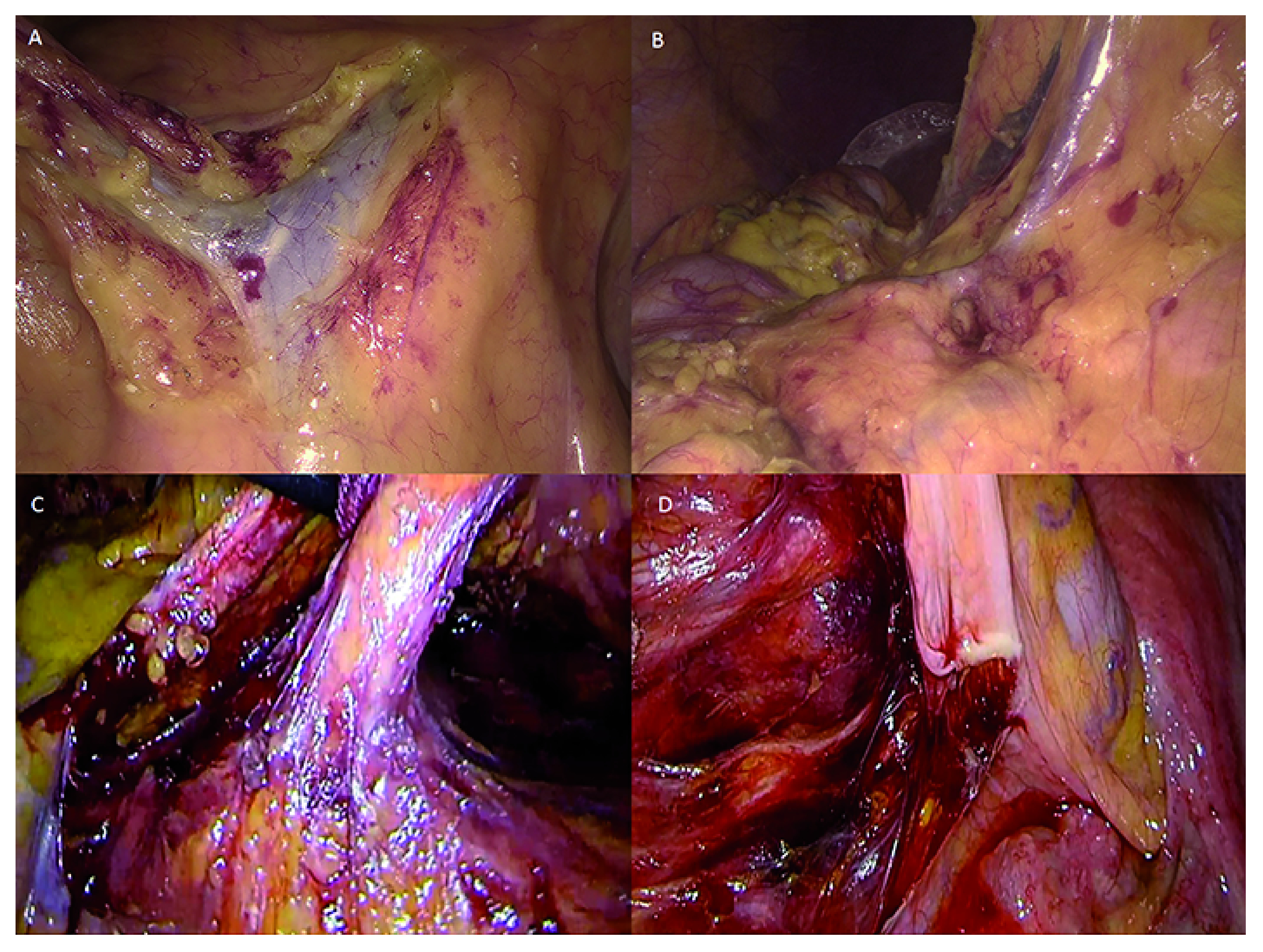
**A**) Ileocolic vein and inferior mesenteric vein (HD technology). **B**) Middle colic vein (4K ultra HD technology). **C**) Gerota’s fascia and inferior mesenteric artery (HD technology). **D**) Hypogastric plexus at the origin of the inferior mesenteric artery (4K ultra HD technology). Images were obtained retrospectively from the video electronic library of our institution, which contains records of all minimally invasive procedures of patients who gave consent for publication of the images for educational or research purposes.

Figure 1A shows the ileocolic vein and the inferior mesenteric vein dissected during a right hemicolectomy with complete mesocolic excision. Figure 1B shows the middle colic vein isolated during the same procedure performed with 4K ultra HD technology. Figure 1C shows the Gerota fascia and the inferior mesenteric artery isolated during a left hemicolectomy with HD technology. Figure 1D shows the hypogastric plexus at the origin of the inferior mesenteric artery spared during anterior rectal resection with 4K ultra HD technology.

## Discussion

The main finding of our study is the statistically significant reduction in surgical time with the 4K ultra HD technology compared to normal HD technology. Intraoperative blood loss was also statistically significantly reduced in patients operated on with 4K ultra HD technology.

4K ultra HD monitors were introduced in our surgical department in January 2018, replacing full HD technology for major colorectal laparoscopic procedures. Accurate visualization of the anatomical structures is crucial during laparoscopic procedures. Recognizing the vascular structures, their divisions and their course within the mesocolic fat tissue is of fundamental importance in oncological surgery ^12^. Lymph node visualization along the course of the vessels and the ability to remove them without encountering troublesome bleeding allow a more precise dissection ^13^.

Cutting edge technologies like 4K ultra HD allow the surgeon to be even more minimally invasive. Abderlahman *et al.* reported on the importance of the quality of 4K ultra HD images in reducing mistakes in structure identification ^1^. Moreover, during left sided colorectal laparoscopic procedures, a precise visualization of the autonomic nerves is related to a better preservation of the genitourinary function. Especially for LRARs, both preservation of the autonomic nerves and avoiding injury to the mesorectum are essential for functional outcomes ^14^.

The results we reported show how, following the introduction of a 4K technology, operating time for the same operations performed by the same surgeons decreased significantly. This may lead to lower operating room costs, as well as to the clinical benefits of a reduction in the peri-operative stress that patients are subjected to due to the duration of surgery and use of anesthesia ^15^. The significant reduction of intraoperative blood loss in the two groups could be related to the improved capability in recognizing vascular structures that 4K ultra HD permits.

Although it was not scientifically investigated in the present work, the subjective impressions of the operating surgeons are important when a new technology is introduced. All participating surgeons subjectively reported a much more detailed image with the 4K ultra HD technology. They all depicted a clearer understanding of the anatomical structures and of the surgical planes. In LRARs above all, the advantages of the 4K ultra HD view have been particularly enhanced, given the complexity of the anatomical field ^16, 17^. All operators reported ending procedures with less eye fatigue using 4K technology, although this could not be measured. The eye fatigue was reported to be minimal during the procedures performed in 2018. This finding is important when related to the fact that in almost all cases, the surgeon has to perform three or four procedures in the same day. Moreover, the 4K ultra HD technology could help in reducing the inevitable eye fatigue that aging brings ^18^.

This study has several biases, beside its retrospective nature. The estimation of the validity of a technology cannot be entrusted only to the operative time required and instead consists of much more complex analysis. However, the reduction in surgical time is a reliable assessment of the technological improvement reached. The subjective reports of the intraoperative perception of the operating surgeons were not structured in intelligible questionnaires. Further efforts should be made to produce a specific tool to investigate the quality of the devices used in mini-invasive surgery.

## Conclusions

4K ultra HD technology applied to colorectal oncologic laparoscopic procedures is safe and effective in reducing surgical time and minimizing intraoperative blood loss compared to normal HD technology.

## Data availability

### Underlying data

Zenodo: 4K Ultra HD Technology Reduces Operative Time And Intra Operative Blood Loss In Colorectal Laparoscopic Surgery. https://doi.org/10.5281/zenodo.3603342 ^9^

This project contains the following underlying data:

- Patients characteristics and surgical outcomes.xls
- Original surgical image files in JPG format

### Extended data

Zenodo: 4K Ultra HD Technology Reduces Operative Time And Intra Operative Blood Loss In Colorectal Laparoscopic Surgery. https://doi.org/10.5281/zenodo.3603342 ^9^

This project contains the following extended data:

- Informed consent.pdf (copy of consent form in Italian)
- Informed consent (english version).pdf (copy of consent form in English)

Data are available under the terms of the Creative Commons Attribution 4.0 International license (CC-BY 4.0).

## Consent

Written informed consent for publication of the patients’ data and clinical images was obtained from each patient involved.
