# Supplementary material for: 4K ultra HD technology reduces operative time and intraoperative blood loss in colorectal laparoscopic surgery
Source: F1000Res. 2020 Feb 11;9:106. [Version 1] doi: 10.12688/f1000research.21297.1 (PMC7400694; doi:10.12688/f1000research.21297.1)
Supplement: Peer review history

# Peer review: Reviewer response for version 1

**DOI:** 10.5256/f1000research.23454.r59927

**Published:** 2020-07-30

**Authors:** Isacco Montroni

**Affiliations:** 1 Colorectal Surgery, Infermi Hospital-Faenza, Faenza, Italy

The abstract is clear and well structured according to the guidelines of the journal.

 The introduction properly explains how technology and surgery have to get along improving the surgical outcomes. However, I think it is crucial that the results provided should be explained in a scientific way. From this point of view, this article is a quite good attempt towards this direction.

 Looking at the extended materials attached data are well collected and presented in a precise way. I believe that having the chance to see the original data is a great opportunity for whoever wants to read an article and therefore I congratulate with the journal and the authors.

 The two cohorts of patients undergoing elective colo-rectal laparoscopic procedures are homogeneous in terms of tumor location, age, and sex and therefore are fully comparable. The authors are assuming that the introduction of new technology could have led to better surgical outcomes. The way they tried to prove it is a retrospective analysis that does not actually provide the best level of evidence. However, when you compare two different types of technologies, developing an RCT is really difficult and it could also be un-ethical.

 Taking a look at the results the authors depicted a reduction in blood loss and surgical time in favour of patients operated with the new technology. This result can be of some interest in its field even though cannot be considered conclusive of the comparison (as stated by the authors in the limitations).

 I think another important issue scoring a point for this trial is that it is not sponsored by the company providing the 4k technology. Usually, this kind of trial comes straight from the pharma. Here instead this bias can be solved by the fact that the authors conducted the study independently and therefore without having pressures from the providing company.

 We do not have to forget that the connection between clinical research and industry has lots of precious  points but also lots of grey zones. The attempt done by the authors is from this point of view of great value and should be taken in serious consideration.

 From a purely practical point of view:

- This study is well understandable.
- Methods are clear and complete.
- Results are well explained and as previously said, they come together with the full amount of data.
- Statistics is good enough.
- The discussion could be implemented but still, there are not so many materials in the literature to compare these results with. This latter also shows how this simple study could be of some value/novelty.
- I consider the study suitable for indexing.

Is the work clearly and accurately presented and does it cite the current literature?

Yes

If applicable, is the statistical analysis and its interpretation appropriate?

Yes

Are all the source data underlying the results available to ensure full reproducibility?

Yes

Is the study design appropriate and is the work technically sound?

Yes

Are the conclusions drawn adequately supported by the results?

Yes

Are sufficient details of methods and analysis provided to allow replication by others?

Yes

Reviewer Expertise:

Colorectal mini-invasive surgery.

I confirm that I have read this submission and believe that I have an appropriate level of expertise to confirm that it is of an acceptable scientific standard.

# Peer review: Reviewer response for version 1

**DOI:** 10.5256/f1000research.23454.r61891

**Published:** 2020-07-29

**Authors:** Matteo Uccelli

**Affiliations:** 1 Department of Surgery, S.I.C.OB. (Italian Society of Bariatric Surgery) Referral Center for Bariatric Surgery, San Donato Group, Policlinico San Marco, Bergamo, Italy

The authors present a retrospective analysis of a cohort of patients operated on for elective laparoscopic procedures for colo-rectal cancer during two different time frames. The main change reported among the two time periods is the introduction of a new visual technology.

 From this point of view, we can call this a “before and after trial”. The information provided is therefore not comparable with an eventual RCT since the introduction of new technology makes the old one un-applicable. Thus the data provided, although coming from a retrospective analysis can be reliable.

 The surgical volume reported are adequate to consider the Unit where the study was performed a “high volume unit” solving the possible bias of the learning curve.

 The main conclusion reached by the authors is that operative times could be reduced by the introduction of a 4K technology. Such a relationship between technology and surgical time is interesting although partial and it is commented by the authors in the limitation assuming that “the estimation of the validity of a technology cannot be entrusted only to the operative time required, but consists of a much more complex analysis. However, the reduction in surgical time is a reliable assessment of the technological improvement reached”.

 Also, the idea of structuring a tool to inquire about the surgeon’s perception when introducing a new technology is relevant and should be taken into consideration for future studies.

 Reduction in intraoperative blood loss in the 4k patients is an important finding even if the incidence of post-operative bleeding is substantially equal in the two groups. From this point of view, the blood loss data correlates more with a precise dissection more than with the complication rate, scoring a point for the 4k technology.

 This trial is simple but clear. It describes the comparison in terms of surgical outcomes of two different visual laparoscopic technology in two similar time frames. It is an interesting attempt to evaluate the relationship between technical means and surgical activity which is oftentimes put aside.

 Statistical analysis are clear and well exposed.

 The background is fluid and contextualized.

 The discussion is clear and it does not assume more than what it should according to the nature of the trial itself. Limitations are reported and clearly understandable.

 As previously reported I think that this trial should be accepted for publication.

Is the work clearly and accurately presented and does it cite the current literature?

Yes

If applicable, is the statistical analysis and its interpretation appropriate?

Yes

Are all the source data underlying the results available to ensure full reproducibility?

Yes

Is the study design appropriate and is the work technically sound?

Yes

Are the conclusions drawn adequately supported by the results?

Yes

Are sufficient details of methods and analysis provided to allow replication by others?

Yes

Reviewer Expertise:

Colorectal laparoscopic surgery

I confirm that I have read this submission and believe that I have an appropriate level of expertise to confirm that it is of an acceptable scientific standard.
